# Genotypic Diversity and Epidemiology of Human Rhinovirus Among Children With Severe Acute Respiratory Tract Infection in Shanghai, 2013–2015

**DOI:** 10.3389/fmicb.2018.01836

**Published:** 2018-08-07

**Authors:** Yanjie Zhao, Jun Shen, Bingjie Wu, Gaoshan Liu, Roujian Lu, Wenjie Tan

**Affiliations:** ^1^Key Laboratory of Laboratory Medicine, Ministry of Education, Institute of Medical Virology, Wenzhou Medical University, Wenzhou, China; ^2^National Institute for Viral Disease Control and Prevention, Chinese Center for Disease Control and Prevention, Beijing, China; ^3^Infectious Disease Department, Children’s Hospital of Fudan University, Shanghai, China

**Keywords:** human rhinovirus, genotype, severe acute respiratory infections, children, epidemiology

## Abstract

Human rhinovirus (HRV), and particularly HRV-C, is increasingly recognized as a cause of severe acute respiratory infections (SARIs). However, little is known about the genotypic diversity and epidemiology of HRV among children with SARI. Thus, we investigated the genotypic diversity and epidemiology of HRV in children with SARI in China over a 2-year period. In total 1,003, nasopharyngeal aspirates were collected from children hospitalized with SARI in Shanghai from 2013 to 2015. HRV was screened for by a PCR method targeting the viral 5′ UTR and was genotyped by sequencing of the VP4–VP2 region of the HRV genome. We also screened for 15 other common respiratory viruses to assess the prevalence of co-infection with HRV. The patient demographic and clinical data were reviewed. HRV was detected in 280 (27.9%) of the 1,003 specimens: HRV-A in 140 (14.0%), HRV-B in 21 (2.1%), HRV-C in 56 (5.6%), and HRV-untyped in 63 (6.3%). A phylogenetic analysis identified 77 genotypes (43 HRV-A, 10 HRV-B, and 24 HRV-C), among which A78, A12, A89, B70, C2, C6, and C24 predominated. HRV-A was detected mainly in winter 2013 and autumn 2014, while HRV-C detection peaked in autumn 2013 and 2014. The detection frequency of HRV-A was highest in patients <5 years old. Most HRV co-infections involved adenovirus, human bocavirus, and/or human respiratory syncytial virus. In conclusion, HRV-A and -C predominate in children with SARI in Shanghai. Among the 77 genotypes detected, A78, A12, A89, B70, C2, C6, and C24 were the most frequent. The HRV species responsible for SARIs differs according to season and age.

## Introduction

Human rhinovirus (HRV), a single-stranded positive-sense RNA virus, belongs to the genus Enterovirus and family Picornaviridae, and is classified as HRV-A, -B, or -C. HRV-A and HRV-B were discovered in the 1950s (Price, 1956), while HRV-C was identified using molecular techniques in 2006 (Lamson et al., 2006; Lau et al., 2007). According to the 2017 International Committee on the Taxonomy of Viruses (ICTV) release^1^, a total of 168 HRV genotypes (80 HRV-A, 32 HRV-B, and 56 HRV-C) are recognized (Kuroda et al., 2015).

Human rhinovirus genomic RNA is approximately 7.2 kb that consisting of a single open reading frame (ORF) encodes 11 proteins, with 5′ and 3′ untranslated regions (UTR) at both end, respectively. The ORF encodes a poly-protein which is cleaved by viral proteases to produce 11 proteins including four structural viral proteins (VP) 1 to 4. Compared to the rest of the HRV genome, the capsid proteins exhibit a high degree of heterogeneity resulting in a wide range of antigenic diversity. RT-PCR assays targeted the 5′-UTR are usually used for HRV clinical detection. HRV species and types are classified almost exclusively now based on VP1 or VP4/VP2 sequence alignments (Wisdom et al., 2009).

Human rhinovirus is a frequently detected respiratory virus in children with mild acute respiratory infection (ARI), but may also lead to more-severe respiratory tract symptoms, such as pneumonia, bronchiolitis, and asthma. HRV is, after respiratory syncytial virus (RSV), the second most frequent viral cause of community-acquired pneumonia and other severe acute respiratory infections (SARIs) (Honkinen et al., 2012; Esposito et al., 2013). HRV-C is more frequently associated with wheezing episodes, asthma exacerbations, and lower respiratory tract infections compared with HRV-A and -B (Linsuwanon et al., 2009; Gern, 2010; Bizzintino et al., 2011). However, there is reportedly no relationship between disease severity and HRV species (Lee et al., 2012; Chen et al., 2015; van der Linden et al., 2016).

Data on the genotypic diversity and epidemiology of HRVs in children with SARI are sparse. Thus, we evaluated the predominant HRV species and genotypes, and their associations with the clinical characteristics, of 1,003 children hospitalized with SARI from 2013 to 2015 in Shanghai, China.

## Materials and Methods

### Ethics Issues

All aspects of the study were performed in accordance with the national ethics regulations and approved by the Ethics Committee of the Children’s Hospital of Fudan University (Jun Shen; CHFU2013016) as well as the Ethics Committee of National Institute for Viral Disease Control and Prevention (RL; IVDC2013022). Participants were received “Written Informed Consent” on the study’s purpose and of their right to keep information confidential. Written consent was obtained from all participants or their guardians.

### Patients and Sample Collection

From June 2013 to August 2015, 1,003 nasopharyngeal aspirates (NPAs) were collected from children hospitalized with SARI in the Children’s Hospital of Fudan University, Shanghai, China. The revised World Health Organization SARI case definition (World Health Organization [WHO], 2011) was adopted and cases with clinical suspicion of SARI for children was enrolled (Zhang et al., 2013; Wang et al., 2016). Eligibility and classification of the clinical syndromes of SARI were determined from individual’s original record of medical history and examination. The criteria of hospitalized patient inclusion were: sudden onset of fever >38°C and cough or sore throat and difficulty breathing (dyspnea, oxygen saturation < 90%). Additional criteria were a normal or low leukocyte count, or lower chest wall indrawing. Demographic data and clinical findings at the time of diagnosis were recorded on a standard form. All NPA samples were stored at -80°C until use.

### HRV Detection and Genotyping

Viral nucleic acid was extracted from 200 μL of sample using QIAamp MinElute Virus Spin Kits (Qiagen, Germany). cDNA was synthesized using an AMV reverse transcriptase and random hexamer primers (Promega, United States), as described previously (Lu et al., 2012). Nested RT-PCR targeting of the 5′-UTR was employed for HRV screening, and of the VP4–VP2 regions for genotyping. All (totally nine, except HEV IS only) of HRV or HRV/HEV primers from 5-UTR to VP4–VP2 were used to detect HRV, as described previously (Wisdom et al., 2009). Specimens from which amplification of the VP4–VP2 regions failed were defined as untyped. PCR products were confirmed by sequencing. Phylogenetic analysis was conducted using Molecular Evolutionary Genetics Analysis (MEGA) software (ver. 7).

### Detection of Common Respiratory Viruses

Human rhinovirus-positive specimens were screened for influenza virus types A and B, parainfluenza virus types 1 to 3, RSV, picornaviruses (enteroviruses and rhinoviruses), and adenovirus (AdV) using three multiple-nested-PCR assays; and for human metapneumovirus (hMPV), human bocavirus (HBoV), and human coronavirus 229E/OC43/NL63/HKU1 using nested-PCR assays. The multiple-nested and nested PCRs were performed as described previously (da Silva Filho et al., 2012; Lu et al., 2012). All PCR products of 15 common respiratory viruses were confirmed by sequencing.

### Statistical Analysis

Data analysis was performed using PASW (ver. 18; SPSS Inc., United States) and GraphPad Prism software (ver. 5.0; GraphPad Software, Inc., United States). Age, maximum body temperature, laboratory parameters, clinical features, and HRV prevalence were compared by χ^2^-test or Fisher’s exact test for categorical variables, and by two-tailed paired Student’s *t*-test for continuous variables. A value of *P* < 0.05 was considered indicative of statistical significance.

## Results

### Epidemiology of HRV

In total, 1,003 NPAs were collected. The male: female ratio was 609:394 (1.55:1) and the median age was 1 year (range: 10 days to 15 years). HRV was detected in 280 (27.9%) of the 1,003 specimens: HRV-A in 140 (14.0%), HRV-B in 21 (2.1%), HRV-C in 56 (5.6%), and HRV-untyped in 63 (6.3%). The HRV detection rates are shown in **Table 1**. The detection rates of HRV-A and -B differed significantly (*P* < 0.001). In general, the 5′-UTR region highly conserved between HRV and enterovirus, causing cross-reactivity in RT-PCR assay for the two viruses. In the current study, 42 enterovirus positive samples also were detected by nested RT-PCR targeting of the HRV 5′-UTR.

**Table 1:** Population demographic of HRV-positive specimens.

Variable	HRV-A (*N* = 140)	HRV-B (*N* = 21)	HRV-C (*N* = 56)	Untyped (*N* = 63)	Total (*N* = 280)
**Gender**					
Male	91 (65%)	14 (66.7%)	34 (60.7%)	44 (69.8%)	184 (65.7%)
Female	49 (35%)	7 (33.3%)	22 (39.3%)	19 (30.2%)	96 (34.3%)
*P*-value	0.263	0.573	1.000	0.126	0.044^∗^
**Age group (years)**					
<1 Year	78 (55.7%)	7 (33.3%)	24 (42.9%)	32 (50.8%)	141 (50.3%)
1–2 Years	33 (23.6%)	7 (33.3%)	14 (25%)	13 (20.6%)	67 (23.9%)
3–4 Years	17 (12.1%)	0	11 (19.6%)	8 (12.7%)	36 (12.9%)
5–9 Years	9 (6.4%)	6 (28.6%)	5 (8.9%)	9 (14.3%)	29 (10.3%)
10–14 Years	3 (2.1%)	1 (4.8%)	2 (3.6%)	1 (1.6%)	7 (2.5%)
*P*-value	0.008^∗^	0.124	0.235	0.810	0.055

*^∗^P < 0.05.*

Human rhinovirus infected significantly more males than females (65.7% vs. 34.3%, *P* = 0.044), and HRV was detected in patients of all ages, although HRV-B was not detected in those aged 3–4 years (**Table 1**). The detection frequency of HRV-A was significantly lower in patients aged 5–9 years (*P* = 0.008), but the frequencies of detection of HRV-B, -C, and -untyped did not differ according to age.

**FIGURE 1:**
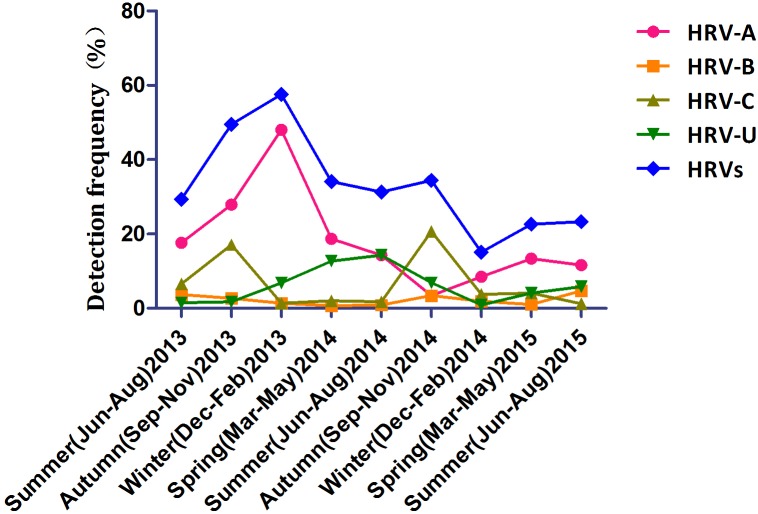
Temporal distribution of the HRV species between June 2013 and August 2015 inclusive.

The seasonal distribution of HRV is shown in **Figure 1**. HRV-A was detected most frequently and peaked in winter 2013 and autumn 2015. HRV-C detection peaked in autumn (17.1% in 2013 and 20.7% in 2014). HRV-B was detected year-round at similar frequencies in all seasons. Detection of HRV-untyped peaked in summer and spring 2014 and was at a low level thereafter.

### Genotypic Diversity of HRV

**FIGURE 2:**
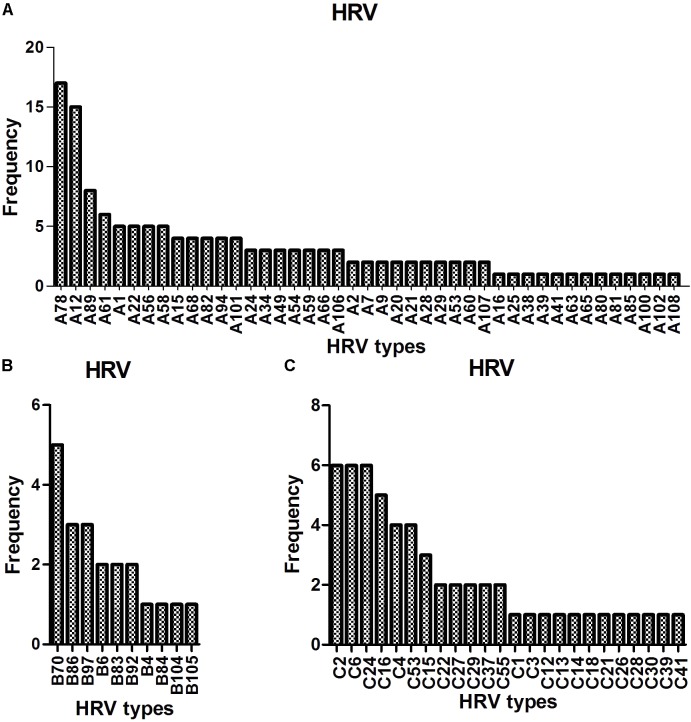
Detection of HRV genotypes in the study **(A–C)**.

Sequence analysis based on 420 bp of the VP4–VP2 region of 217 of 280 the HRV strains yielded 77 genotypes: 43 HRV-A (most frequently detected: -A78 [17/140, 12.1%], followed by -A12 [15/140, 10.7%], -A89 [8/140, 5.7%], -A61 [*N* = 6], and -A1, -A22, -A56, and A-58 [*N* = 5 each]), 10 HRV-B (most frequently detected: -B70 [5/21, 23.8%], followed by -B86 and -B97 [3/21, 14.3%]) and 24 HRV-C (most frequently detected: -C2, -C6, and -C24, [6/56, 10.7%], followed by -C16 [5/56, 8.9%] and -C4 and -C53 [4/56, 7.1%]) (**Figure 2**).

### Clinical Characteristics

The clinical characteristics of the patients with HRV infections are listed in **Table 2**. All HRV-positive patients presented with SARI. Fever, cough, gasping, cyanosis, and diarrhea were the most common symptoms at presentation. There was no significant difference in clinical symptoms according to HRV species, except that cough and cyanosis were less frequent in patients infected with HRV-A and HRV-C, respectively (*P* = 0.048 and 0.005).

**Table 2:** Clinical characteristics of patients with HRV infections in the study.

Characteristics	HRV-A (*N* = 140)	HRV-B (*N* = 21)	HRV-C (*N* = 56)	HRV-Untyped (*N* = 63)	*P*-value
Fever (mean/median)	39.0/39.0	39.2/39.0	39.1/39.0	38.7/39.0	
Cough	119 (85%)	21 (100%)	52 (92.8%)	56 (88.9%)	0.048^∗^
Gasping	53 (37.9%)	8 (38.1%)	23 (41.1%)	22 (34.9%)	0.924
Cyanosis	17 (12.1%)	3 (14.3%)	0	7 (11.1%)	0.005^∗^
Diarrhea	17 (12.1%)	1 (4.8%)	4 (7.1%)	11 (17.5%)	0.228
Bronchitis	9 (6.4%)	1 (5%)	9 (16.1%)	5 (7.9%)	0.206
Bronchial asthma	2 (1.4%)	0	1 (1.8%)	2 (3.2%)	0.699
Pneumonia	95 (67.9%)	16 (76.2%)	37 (66.1%)	41 (65.1%)	0.804
Severe pneumonia	15 (11.0%)	4 (19.0%)	4 (7.1%)	9 (14.3%)	0.435
Respiratory failure	11 (7.1%)	1 (4.8%)	6 (10.7%)	7 (11.1%)	0.732
Death	0	0	0	2 (3.2%)	0.111

*^∗^P < 0.05.*

### Co-infection With Other Respiratory Viruses

Of the 280 HRV-positive patients, 131 (46.8%) were co-infected—102 with one other virus, 21 with two other viruses, and 8 with three other viruses (**Table 3**). Most such co-infections involved AdV, HBoV, and RSV (39.7, 38.2, and 17.6%, respectively). The patients with co-infections did not have more serious disease (data not shown).

**Table 3:** Co-detections of HRV and other respiratory virus in the study.

2 Virus (*N* = 102)		3 Viruses (*N* = 21)		4 Viruses (*N* = 8)	
HRV+ADV	36	HRV+ADV+HBoV	7	HRV+ADV+HBoV+HCoV	2
HRV+HBoV	32	HRV+HBoV+hRSV	3	HRV+PIV+HBoV+ENV	1
HRV+hRSV	16	HRV+ADV+hRSV	2	HRV+PIV+ADV+ENV	1
HRV+PIV	7	HRV+ADV+PIV	1	HRV+PIV+HBoV+ADV	1
HRV+HCoV	7	HRV+ADV+ENV	1	HRV+hRSV+HBoV+ADV	1
HRV+FluA	2	HRV+ADV +HCoV	1	HRV+FluA+ADV+hRSV	1
HRV+ENV	2	HRV+HBoV+PIV	1	HRV+HMPV+HBoV+ ENV	1
HRV+HMPV	1	HRV+HBoV+HCoV	1		
		HRV+hRSV+HCoV	1		
		HRV+FluA+HMPV	1		
		HRV+FluA+HBoV	1		
		HRV+ENV +HCoV	1		

*HRV, human rhinovirus; ADV, adenovirus; HBoV, human bocavirus; hRSV, human respiratory syncytial virus; PIV, parainfluenza virus; HCoV, human coronavirus; FluA, influenza type A; ENV, enterovirus; HMPV, human metapneumovirus.*

## Discussion

We evaluated the HRV genotype distribution in children with SARI in the Children’s Hospital of Fudan University, Shanghai, China from June 2013 to August 2015. Of the 1,003 samples, 280 (27.9%) were positive for HRV (HRV-A, 50.0%; HRV-B, 7.5%; HRV-C, 20%; and HRV-untyped, 22.5%). This HRV detection rate is consistent with prior studies (11.0–40.6%) worldwide, as are the proportions of the three HRV species (HRV-A, 44.4–56%; HRV-B, 2–12%; and HRV-C, 25–45.3%) (Xiang et al., 2010; Henquell et al., 2012; Rahamat-Langendoen et al., 2013; Marcone et al., 2014; Tsatsral et al., 2015; Xiao et al., 2015; Milanoi et al., 2016; van der Linden et al., 2016; Blaschke et al., 2018). This prevalence of HRV-untyped is higher than in a recent study in Chongqing (13%) (Xiao et al., 2015), perhaps because our study involved patients with SARI. We achieved identical results using another nested-PCR detection primer also targeting the VP4–VP2 region (data not shown). Further research on whether these HRV-untyped contain new genotypes is warranted.

We detected 77 HRV genotypes in Shanghai from 2013 to 2015 (43 HRV-A, 10 HRV-B, and 24 HRV-C). The predominant HRV-A genotypes were A-78, A-12, A-89, and A-61; those of HRV-B were B-70, B-86, and B-97; and the predominant HRV-C genotypes were C-2, C-6, C-24, and C-16. Variation in the prevalent HRV genotype has been reported by others. In Amsterdam, 129 HRV genotypes were detected in inpatients and outpatients: A-12, A-78, and C-2 predominated (van der Linden et al., 2016). In Buenos Aires, 30 HRV genotypes were detected in children with acute respiratory infections: A-101, A-49, and C-10 predominated (Marcone et al., 2014). In Asia, 59, 36, and 28 HRV genotypes were detected in Mongolia, Beijing, and Cambodia, respectively: A-46, A-12, A-78, B-79, B-86, C-2, and C-36; A-12 and B42; and A-89, A-78, B-79, and C-6, respectively, predominated (Xiang et al., 2010; Naughtin et al., 2015; Tsatsral et al., 2015). Therefore, a large number of HRV genotypes circulate simultaneously, and some genotypes (such as A-12, A-78, and C-2) are the more prevalent types across worldwide although various predominant genotype patterns were reported geographically.

Human rhinovirus circulated throughout this 2-year study with peaks in winter 2013 and autumn 2014; HRV-C predominated in autumn. This seasonal pattern is similar to those reported previously (Xiang et al., 2010; Marcone et al., 2014; van der Linden et al., 2016). Also, HRV-A was detected most frequently in those <5 years old, while the frequency of detection of HRV-B and -C did not differ by age. In contrast, in Mongolia, HRV-A and -C are detected more frequently in younger children, and -B more frequently in older children (Tsatsral et al., 2015).

Human rhinovirus-A and HRV-C are reportedly associated with more severe illness (Miller et al., 2011; Fawkner-Corbett et al., 2016); however, others have reported no link between species and disease severity (Xiang et al., 2010; Henquell et al., 2012; Rahamat-Langendoen et al., 2013; Marcone et al., 2014). In this study, there were no significant differences in clinical symptoms among the three HRV species, except that cough and cyanosis were less frequent in patients with HRV-A and HRV-C, respectively. HRV-C infection in boys under 5 years old with acute asthma significantly increases the risk of moderate/severe exacerbations (Lambert et al., 2017). Moreover, of 570 HRV-positive hospitalized patients with community-acquired pneumonia, 57 (10%) had viremia; the vast majority (98.2%) of viremic patients were infected with HRV-C. The frequency of HRV viremia was higher in patients 1–2 years of age, and patients with viremia were more likely to have severe clinical symptoms, such as chest retraction, wheezing, and asthma (Lu et al., 2017). Therefore, particular HRV species may be associated with disease severity at specific situations which further research is warranted.

Almost half (46.8%) of the HRV-positive patients were co-infected with other respiratory viruses, most frequently ADV, HBoV, and hRSV. This may reflect the viruses in circulation at the time, consistent with previous reports (Xiang et al., 2010; van der Linden et al., 2016; Blaschke et al., 2018). Additionally, for those patients infected with 2–4 respiratory viruses, further research is necessary to demonstrate the dominant virus by qPCR positive with CT value and impact on disease severity.

In summary, we report for in detail the first time the variety of HRV genotypes in circulation and their distribution according to season and age group, as well as their clinical symptoms, in children hospitalized with SARI in China during a 2-year period. Our data shown that HRV was detected in 280 (27.9%) of the 1,003 NPAs from children with SARI in Shanghai, in which HRV-A and -C were predominate. Among the 77 HRV genotypes detected, A78, A12, A89, B70, C2, C6, and C24 were the most frequent. The HRV species responsible for SARIs differs according to season and age.

## Author Contributions

RL, JS, and WT conceived and designed the experiments. YZ, RL, JS, BW, and GL performed the experiments. RL, JS, and WT analyzed the data. RL and WT wrote the manuscript.

## Conflict of Interest Statement

The authors declare that the research was conducted in the absence of any commercial or financial relationships that could be construed as a potential conflict of interest.

## Footnotes

1 http://ictvonline.org/virusTaxonomy.asp
